# Temperature, season, and latitude influence development-related phenotypes of Philippine *Aedes aegypti* (Linnaeus): Implications for dengue control amidst global warming

**DOI:** 10.1186/s13071-022-05186-x

**Published:** 2022-03-05

**Authors:** Frances Edillo, Rhoniel Ryan Ymbong, Alyssa Angel Bolneo, Ric Jacob Hernandez, Bianca Louise Fuentes, Garren Cortes, Joseph Cabrera, Jose Enrico Lazaro, Anavaj Sakuntabhai

**Affiliations:** 1 Mosquito Research Laboratory, Biology Department, University of San Carlos-Talamban Campus, Cebu City, Philippines; 2 National Institute of Molecular Biology and Biotechnology, University of the Philippines Diliman, Quezon City, Philippines; 3 Functional Genetics of Infectious Diseases Unit, Institut Pasteur, Paris, France; 4 Centre National de la Recherche Scientifique, 75015 Paris, France

**Keywords:** *Aedes aegypti*, Dengue, Development-related phenotype, Global warming, Pharate larvae, Hatching rate, Reproductive output

## Abstract

**Background:**

Dengue is endemic in the Philippines. *Aedes aegypti* is the primary vector. This study aimed to determine the hatching behavior and viability of *Ae. aegypti* first-generation (F1) eggs when exposed to temperature and photoperiod regimes under laboratory conditions.

**Methods:**

Parental eggs were collected from selected highland and lowland sites in the Philippine big islands (Luzon, Visayas, and Mindanao) during the wet (2017–2018) and dry (2018) seasons. F1 egg cohorts were exposed separately in environmental chambers at 18, 25, and 38 °C with respective photoperiods for 6 weeks. Phenotypes (percent pharate larvae [PPL], hatch rates [HRs], and reproductive outputs [ROs]) were determined.

**Results:**

Results of multivariate analyses of variance (MANOVA) between seasons showed significant main effects of temperature, season, and big island on all phenotypes across all sites. Significant interaction effects between seasons on all phenotypes across sites were shown between or among (1) season and big island, (2) season and temperature, (3) big island and temperature, (4) season, big island, and temperature, (5) big island, altitude, and temperature, and (6) season, big island, altitude, and temperature. Factors associated with the big islands might include their ecology, available breeding sites, and day lengths due to latitudinal differences, although they were not measured in the field. MANOVA results within each season on all phenotypes across sites showed (1) significant main effects of big island and temperature, and (2) significant interaction effects between big island and temperature within the wet season and (3) between temperature and photoperiod within the dry season. PPL were highest at 18 °C and were formed even at 38 °C in both seasons. Pharate larvae might play an adaptive role in global warming, expanded distribution to highlands, and preponderance to transmit human diseases. HRs in both seasons were highest at 25 °C and lowest at 38 °C. ROs were highest at 25 °C in the wet season and at 18 °C in the dry season.

**Conclusions:**

Temperature and latitude of Philippine big islands influenced the development-related phenotypes of *Ae. aegypti* in both seasons. The two seasons influenced the phenotypes and their interaction effects with big island and/or temperature and/or altitude. Recommendations include year-round enhanced 4S control strategies for mosquito vectors and water pipeline installation in rural highlands.

**Graphical Abstract:**

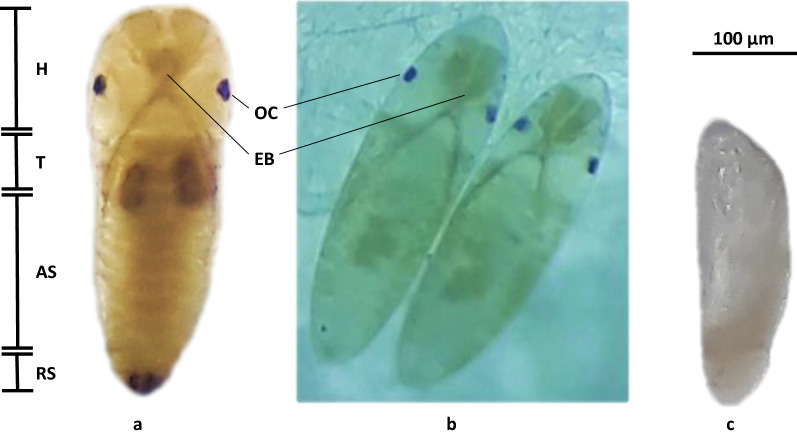

**Supplementary Information:**

The online version contains supplementary material available at 10.1186/s13071-022-05186-x.

## Background

*Aedes aegypti* (Linnaeus), a mosquito vector of dengue, Zika, and chikungunya viruses, occurs widely in the tropics and subtropics. Climate change affects mosquito survival, dispersion, and dengue transmission rates [1]. The macroclimate determines the global range limit of this species, and much of its range in temperate and subtropical regions is sustained by artificial environments [2]. This implies that if domestic environments are available in newly suitable areas, the distribution of *Ae. aegypti* may expand considerably in the near future. In Asia, the Philippines has ranked first in the number of dengue deaths (63), with a total of 17,630 cases as of March 27, 2021 [3]. Direct medical costs of dengue cases amounted to $345 million (in 2012 US dollars) [4]. Dengue outbreaks occurred in temperate regions such as Tokyo, Japan, in 2014, where local transmission by *Ae. albopictus* was recorded after 70 years without confirmed cases [5]; in Madeira, Portugal, with its first dengue epidemic (2187 reported cases) in 2012 by *Ae. aegypti* [6]; and in Baguio city, Philippines, with an increase of over 344% from 385 cases in 2014 to 1710 cases in 2015 [7]. The mosquito’s adaptive behaviors support the prevailing view that a future warmer climate will lead to larger mosquito populations and an increase in dengue transmission [8, 9]. An expanded distribution of *Ae. aegypti* to a previously non-endemic and mountainous region calls for urgent actions to protect public health [10].

Dormancy is an adaptive mechanism that allows some species to withstand harsh environmental conditions through diapause and quiescence [11, 12]. Diapause is genetically programmed, neurohormone-mediated, and the primary mechanism for survival as shown by *Ae. albopictus* and *Culex pipiens* in environments with seasonal change. Quiescence is an irregular dormancy with slowed metabolism resulting from unfavorable environmental conditions that impede larval hatching [13], but is neither a previously programmed event nor hormonally controlled [14]. Developmental arrest is temporary and is immediately reversible. *Aedes aegypti* has been reported to undergo embryonic quiescence but several studies have erroneously reported it as diapause [15, 16]. Diminishing expression of genes associated with lipid storage over time during diapause in *Ae. albopictus* primarily distinguishes between early diapause and quiescence, and this is likely to reflect a physiological convergence of diapause towards quiescence [11]. Moreover, identifying adaptive genes and genetic loci linked to these genes is the first step towards understanding the mechanisms that enable mosquitoes to survive under ecological conditions influenced by global warming. Elucidating the mechanisms that enable them to persist through challenging ecological conditions provides insights that can help predict population dynamics, the trajectory of population expansion, and the occurrence of dengue outbreaks.

Various vector and health-control interventions have resulted in temporary interruptions of disease transmission and have helped reduce the dengue burden. The enhanced 4S strategy of the Philippine Department of Health (DOH) has been the main focus for dengue prevention and control, where “4S” stands for the following: (1) seek and destroy mosquito-breeding sites, (2) seek early consultation if one develops dengue-associated symptoms, (3) employ self-protection measures such as wearing long pants and long-sleeved shirts, and (4) say “no” to indiscriminate fogging, and implement fogging only during outbreaks in hotspot areas [17]. We hypothesized that temperature, season, and latitudinal differences as influenced by photoperiod and altitude might play a role in the development-related phenotypes of *Ae. aegypti* eggs. This study aimed to assess the hatching behavior and viability of *Ae. aegypti* eggs (first-generation, F1) whose parental eggs were collected from selected highland and lowland sites in the country’s big islands (Luzon, Visayas, and Mindanao) during the wet (2017–2018) and dry (2018) seasons, and which had been exposed separately to different temperature and photoperiod regimes under laboratory conditions.

## Methods

### Study sites

**Fig. 1:**
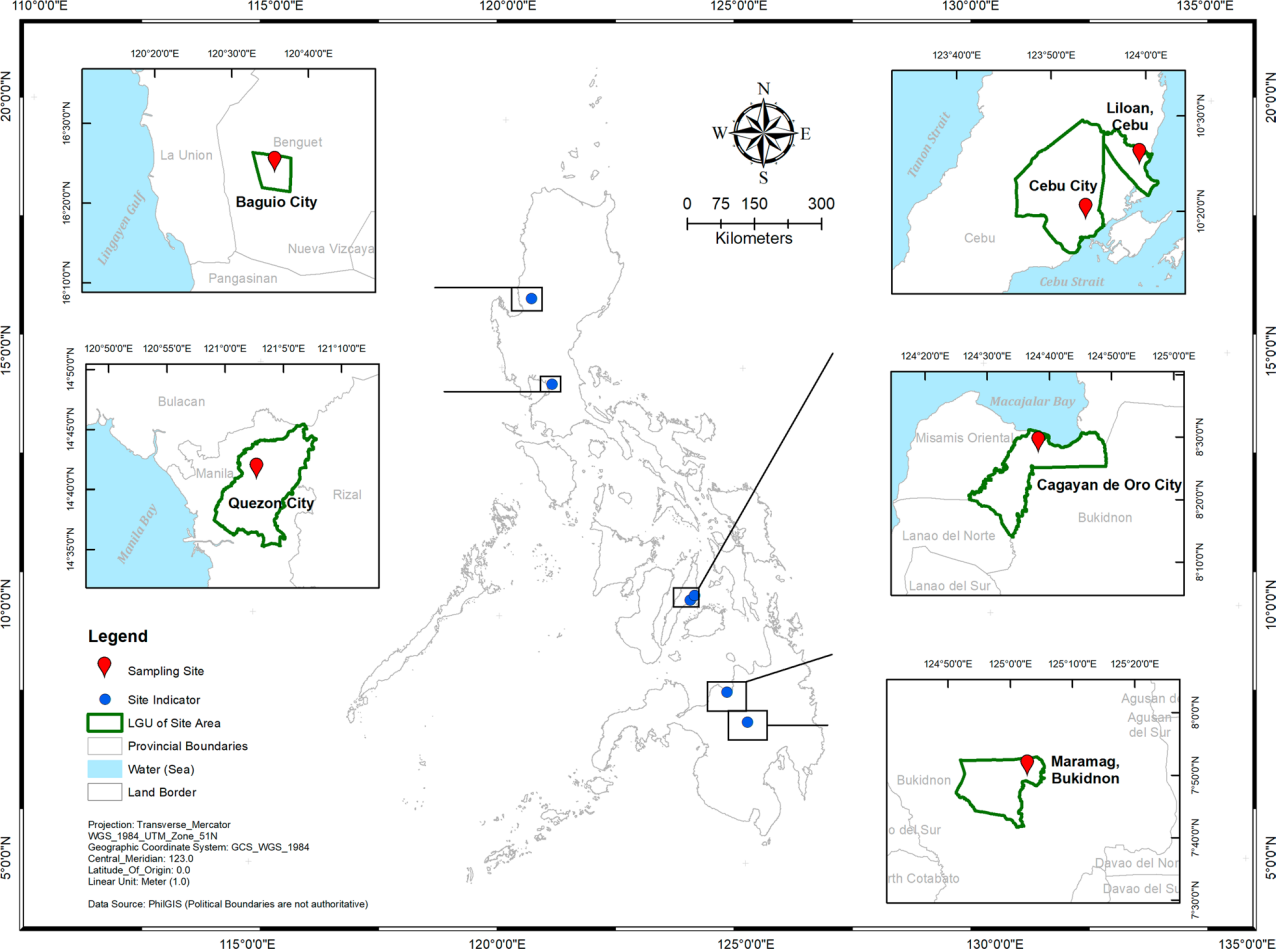
The study sites in Luzon (Baguio and Quezon cities), Visayas (Cebu city mountains and Liloan, Cebu) and Mindanao (Maramag, Bukidnon and Cagayan de Oro city)

**Fig. 2:**
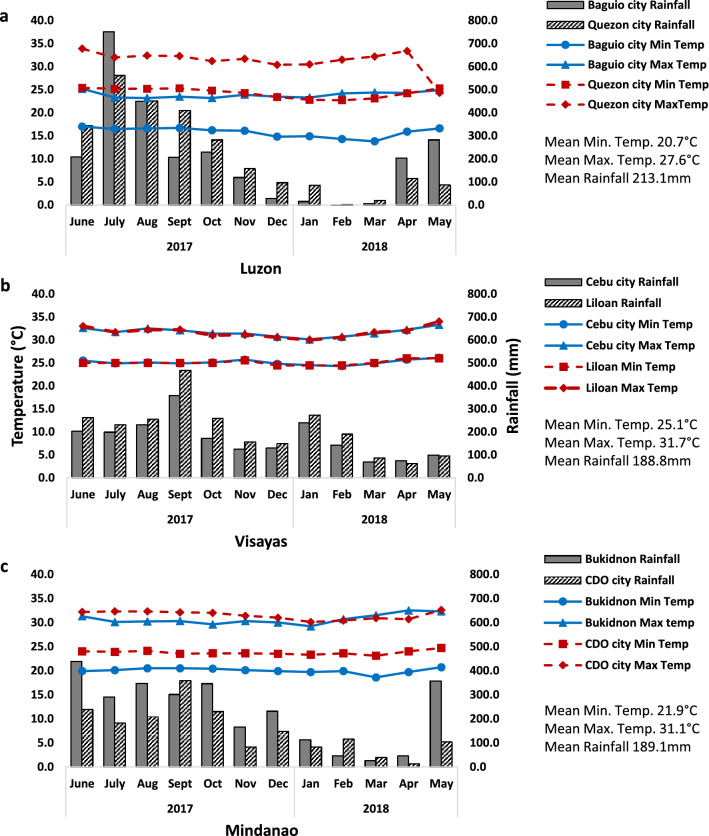
Monthly temperatures (minimum and maximum, °C) and monthly rainfall (mm) for Baguio and Quezon cities (**a**), Cebu city mountains and Liloan, Cebu (**b**), and Bukidnon and Cagayan de Oro city (**c**) from June 2017 until May 2018

We established a two-site category in each Philippine big island, namely lowlands for study sites < 100 m above sea level (m ASL) and highlands for sites > 350 m ASL, considering different ecological topologies across big islands and logistics for mosquito collections. Highland and lowland study sites were selected in each of the Philippine big islands (Fig. 1), with three sub-sites per site, which were located between 0.5 and 3 km apart (Table 1). Highland sites in Luzon, Visayas, and Mindanao included Baguio city (BG); *barangays* (the smallest government unit) in Cebu city (CC) mountains; and Maramag, Bukidnon (BUK), respectively. Lowland sites included Quezon city (QC); Liloan (LIL), Cebu; and Cagayan de Oro city (CDO), respectively. These sites were chosen based on (1) elevation, (2) range of latitudes represented by the big islands, (3) dengue incidence, and (4) similar biotype according to modified Corona’s climate classification [18]. The country has a tropical climate characterized by two seasons, a relatively wet season (June to February) and a dry season (March to May) with longer days and short nights; summer solstice occurs in late June. Meteorological conditions of study sites were taken from the nearest station of the Philippine Atmospheric, Geophysical, and Astronomical Services Administration (PAGASA) and from AccuWeather [19, 20] for Liloan, Cebu (Fig. 2).

**Table 1:** Philippine highland and lowland study sites in Luzon, Visayas, and Mindanao with coordinates, sub-sites, and their elevations with a sample size of *Ae. aegypti* F1 egg cohorts per season

Sites	Coordinates	Sub-sites (elevation m ASL^a^)	Sample size season	
			Wet	Dry
*Luzon*				
Baguio city (BG)	16.402°N; 120.596°E	A. UP-Baguio^b^ (1489)	90	12
		B. Trancoville (1417)	78	12
		C. Cabinet Hill (1474)	76	12
Quezon city (QC)	14.676°N; 121.044°E	A. NIMBB, UPD^c^ (75)	89	12
		B. Daan Tubo (65)	90	12
		C. Payatas B (62)	90	12
*Visayas*				
Cebu city mountains (CC)	10.317°N; 123.891°E	A. Taptap (662)	90	12
		B. Babag 2 (405)	90	12
		C. Tabunan, Cantipla (873)	90	12
Liloan, Cebu (LIL)	10.4121°N; 123.986°E	A. Poblacion (17)	90	12
		B. Yati (21)	90	12
		C. Catarman (16)	90	12
*Mindanao*				
Maramag, Bukidnon (BUK)	7.8592°N; 125.0515°E	A. Lumbo (458)	90	12
		B. Sentro, Dologon (371)	90	12
		C. Musuan, Dologon (386)	90	12
Cagayan de Oro city (CDO)	8.454°N; 124.632°E	A. Gusa (14)	90	12
		B. Cugman (21)	90	12
		C. Bugo (20)	90	12

^a^Meters above sea level

^b^University of the Philippines–Baguio

^c^National Institute of Molecular Biology and Biotechnology, University of the Philippines–Diliman

### Mosquito collections

*Aedes* parental eggs were collected using a modified black plastic ovicidal/larvicidal (O/L) trap or ovitrap (Department of Science and Technology, Manila) method for Mindanao and Luzon sites. Larvae and pupae were collected by a net method [21] from breeding sites in Visayas sites during the wet season (2017–2018); parental eggs were collected by a modified O/L trap method in the dry season (2018). Briefly, the O/L trap consisted of a filter paper-wrapped wooden paddle put diagonally inside a black plastic tumbler filled with tap water; the filter paper served as a substrate for female *Aedes* to lay eggs on. Thirty to 40 O/L traps were placed randomly outside human dwellings under the roof gutters in each sub-site, and were inspected daily for possible oviposition of *Aedes* eggs on the filter paper (henceforth called “egg paper”). Each egg paper was air-dried and put inside a plastic cup for shipment, with permission from the Philippine Bureau of Quarantine, Cebu city.

### Rearing and coupling of *Ae. aegypti* for F1 egg cohort collections

Parental *Aedes* eggs, larvae, and pupae were reared in the insectary at 23–25 °C, 75–80% relative humidity (RH), and respective photoperiods described below for different experiments. Parental eggs were placed in plastic transparent cups and were submerged in an ascorbic acid solution (100 mg of ascorbic acid per liter of distilled water [DW]) for 3 days, which facilitated rapid egg hatching via deoxygenation of the water [22]. Emerged larvae were segregated into several plastic cups (< 100 larvae per cup) containing DW and covered with fine-mesh cloth. The larvae were fed daily with powdered fish food (Sakura; All Aquariums Co., Ltd. Bangkok, Thailand); water was replaced every other day or as needed to prevent accumulation of bacterial scum. Adult mosquitoes were then sorted out by sex and species after eclosion because sometimes *Ae. albopictus* samples were present. Male and female *Ae. aegypti* collected from the same sub-site and sampling date were coupled by placing them in a fine-mesh cloth-covered plastic cup with a filter paper at its bottom.

Single mosquitoes were placed in similar cups as a replacement whenever a partner in a couple died. A cotton ball soaked in 10% sucrose solution was put on top of the fine-mesh cloth. Mosquito couples were allowed to mate over 3–5 days. The mated females were then starved for 24 h, then fed chicken blood mixed with ethylenediaminetetraacetic acid (EDTA) as anticoagulant using a plastic bottle, in which the chicken blood was placed on its bottom depression and sealed with parafilm membrane. The plastic bottle was placed in upright position on top of the mosquito cups covered with fine-mesh cloth with starved couples. Lukewarm water was frequently added inside the plastic bottle to simulate body heat and to attract the females to suck blood. This blood-feeding method is modified from that of Costa-da-Silva et al. [23] to artificially feed *Ae. aegypti* with blood. After feeding, a cotton ball soaked with 10% sucrose solution was again placed on the mesh cloth, and the filter paper at the bottom of the mosquito cup was moistened with DW for the female to lay fertilized eggs [24] ~ 2–5 days later. Each dried egg paper was stored inside a parafilm-sealed plastic cup and put inside a dark cabinet for 1.75–2 months, the conventional storage period for *Ae. aegypti* eggs at 80% hatch rate (HR) [25, 26]. At least 30 morphologically viable eggs from each F1 egg cohort were examined under a light microscope. Viable eggs were smooth, shiny black, ovoid-shaped, and ~ 1 mm long [27], whereas nonviable ones appeared deflated, cracked, or folded.

### Experimental design for wet season-collected *Ae. aegypti*

**Fig. 3:**
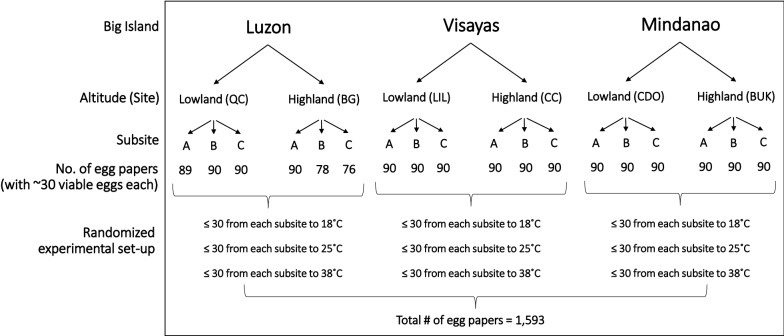
Flowchart summarizing the randomized experimental setup for the wet season *Ae. aegypti* F1 egg papers. Sub-sites for lowland and highland sites are listed in Table 1

Collected parental eggs of *Ae. aegypti* were reared up to adulthood inside the insectary under 12L:12D photoperiod. F1 egg cohorts from the parental generation were individually placed in loosely covered Falcon tubes, which were then exposed separately to regulated temperature regimes (18, 25, and 38 °C) inside their respective environmental chambers (BPIT-407GC model; BP Integrated Technologies Inc., Laguna, Philippines) with RH of 65–75% [28] and photoperiod of 8.5L:15.5D at 225–745 lx for 6 weeks. The photoperiod served to simulate the estimated average number of hours that eggs in different artificial containers were exposed to daylight in the sites during the wet season. Temperature regimes (18, 25, and 38 °C) were based on approximate average cold (18 °C) and extreme hot temperature (38 °C) with average optimum (25 °C) as control based on seasonal months of PAGASA (2017) readings nearest to the sites. A completely randomized design (CRD) was employed; F1 egg cohorts were randomly selected for experimental treatment (i.e., induction of quiescence) of separate temperature regimes (Fig. 3). These environmental chambers were monitored twice daily to ensure their respective conditions throughout the experimental period.

The required sample size for F1 egg cohorts (*n* = 540; i.e., 90 egg cohorts from each site with ~ 30 egg cohorts from each sub-site per temperature regime per season) exposed separately to each temperature regime in an environmental chamber (Bio-Integrated Philippines Inc., Manila) was calculated using G*Power sample size software to provide a statistical power of 80% to detect significant differences in their phenotypes given two altitudes from each big island and three temperature regimes (Table 1) [29].

The F1 egg cohorts were then treated with ascorbate medium (100 mg of ascorbic acid per 1000 ml of DW) inside their respective chambers for 3 days in order to deoxygenate the water and to suppress egg dormancy. Then, they were treated with sodium hypochlorite (NaOCl) solution (50%) at room temperature and observed under a compound light microscope attached to a monitor to determine the number of unhatched embryonated eggs (i.e., pharate larvae) and unhatched incomplete embryos [22, 30, 31]. The presence of a pair of ocelli, egg burster, and abdominal segments of the unhatched embryonated eggs served as indicators of complete embryogenesis, and hence pharate larvae [31]. The phenotypes included percent pharate larvae (PPL); reproductive output (RO), calculated as the number of F1 egg cohorts that produced surviving larvae; and hatch rate (HR), determined with the following formula [22]:

$$\mathrm{HR}=\frac{\mathrm{hatched\, eggs}}{(\mathrm{embryonated\, unhatched\, eggs}+\mathrm{hatched\, eggs})} \times 100.$$

### Experimental design for dry season-collected *Ae. aegypti *

**Fig. 4:**
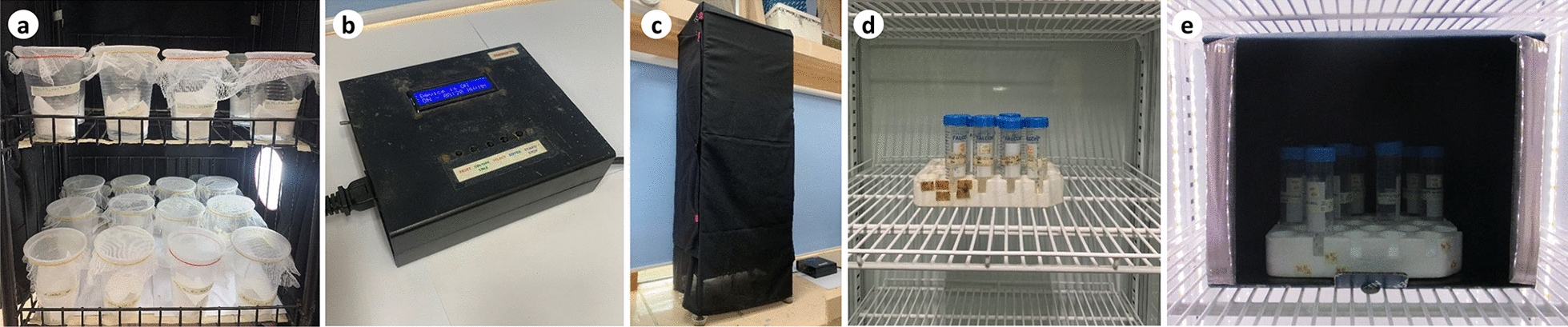
** a–e** Photoperiod setup. Adult *Ae. aegypti* couples inside plastic cups covered with fine-mesh cloth were exposed at 13.5L:10.5D with the use of a lamp **a** programmed by a timer (**b**), within a rack covered with a black cloth (**a**, **c**) inside the insectary. F1 *Ae. aegypti* egg cohorts were exposed inside an environmental chamber programmed at a temperature regime, 65–75% RH, 8.5L:15.5D (control) (**d**) and 12L:12D photoperiod (**e**)

**Fig. 5:**
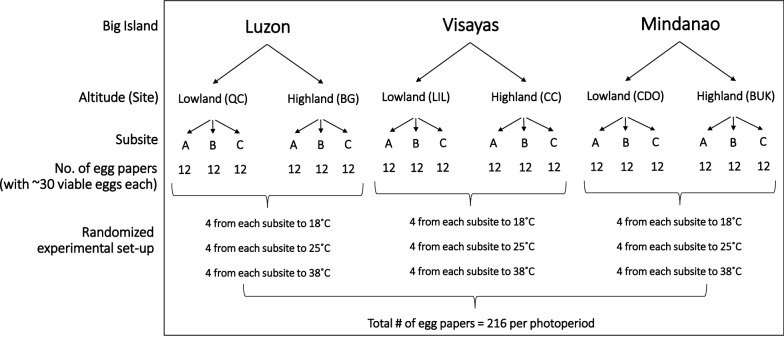
Flowchart summarizing the randomized experimental setup for the dry season *Ae. aegypti* F1 egg papers. Sub-sites for lowland and highland sites are listed in Table 1

Rearing conditions for parental adults collected in the dry season were the same as for those in the wet season. However, there were two groups of samples exposed to photoperiods at 12L:12D (control) and 13.5L:10.5D (experimental) (Fig. 4) based on the average day lengths in the wet and dry seasons, respectively, of PAGASA (2017) records nearest the study sites. Four F1 egg cohorts (each with ~ 30 morphologically viable eggs on a filter paper) of dry season-collected parental eggs from each sub-site for each photoperiod (control at 8.5L:15.5D; experimental at 12L:12D) were separately exposed at 18 °C, 25 °C (control), and 38 °C (*n* = 72 egg cohorts at each temperature and photoperiod) inside environmental chambers for 6 weeks employing CRD (Fig. 5). The experimental photoperiod served to simulate the estimated average number of hours that eggs in different artificial containers were exposed to light in the study sites during the dry season. The required sample size for F1 egg cohorts was calculated using G*Power sample size software to provide a statistical power of 83% in order to detect significant differences in PPL, HRs, and ROs given two altitudes from each big island and three temperature regimes (Table 1) [29]. The F1 egg cohorts were then hatched following the same hatching protocol for parental eggs but without the drying-rehatching step. The egg papers were examined under the microscope to count the larvae 3 days after the eggs hatched, after which the unhatched eggs were bleached using 30% NaOCl solution to expose the pharate larvae. The number of hatched eggs and pharate larvae were recorded for each egg cohort. HRs, PPL, and ROs were calculated.

### Statistical analyses

Data sets for dependent variables (PPL, HRs, and ROs) for both seasons were subjected to full-factorial multivariate analyses of variance (MANOVA) using a generalized linear model (GLM) in order to determine the main and interaction effects of independent variables (i.e., altitude, big island, temperature, photoperiod and/or season) on each dependent variable (PPL, HR, and RO) within and between seasons. Univariate analyses were done after MANOVA’s significant interaction effects, then Tukey’s post hoc tests followed.

## Results

This is the first study on the development-related phenotypes of tropical *Ae. aegypti* collected from different altitudes across the Philippine big islands with latitudinal differences (Luzon at 14.6°N–16.4°N, Visayas at 10°N, and Mindanao at 8°N) (Table 1).

### Development-related phenotypes of wet season-collected *Ae. aegypti*

**Fig. 6:**
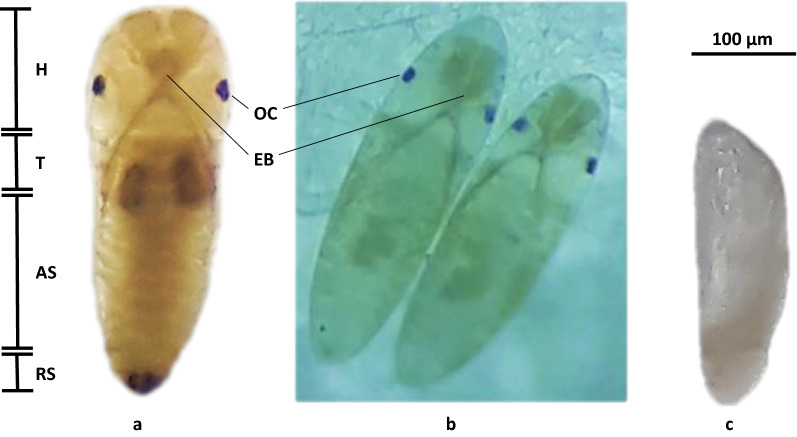
Morphology of pharate larvae (dorsal view) (**a**, **b**) and unhatched incomplete embryo (**c**) of *Aedes aegypti* obtained from this study. Body regions of the pharate larvae are labeled, namely, the head (H), thorax (T), abdominal segments (AS), and respiratory siphon (RS), as well as the ocelli (Oc) and egg burster (EB)

Figure 6 shows the morphological features diagnostic of pharate larvae inside the chorion, which include egg burster, a pair of ocelli, and abdominal segments (a, b), consistent with temperate *Ae. albopictus* and *Ae. aegypti* [22, 31] and the incompletely embryonated eggs that are unable to adjust to their environment (c). Pharate larvae inside the egg chorion completed embryogenesis but did not hatch when externally stimulated by ascorbate, suggesting they were in quiescence towards the end of embryogenesis [22, 32–34].

Across the big islands during the wet season, the overall mean PPL (4.61%) (Table 2), HRs (7.68%), and ROs (4.58) were lowest at 38 °C. Overall mean HR (38.25%) and RO (23.44) were highest at 25 °C, whereas mean PPL were highest at 18 °C (9.79%). Within the big islands, the highest mean PPL (Table 2) were recorded at 18 °C for Luzon (15.73%) and Visayas (7.3%), and at 25 °C for Mindanao (6.79%). For Luzon and Visayas, the highest mean HR (57.01% and 27.44%, respectively) and RO (26.67 and 20.17, respectively) were recorded at 25 °C. Mindanao had the highest mean HR (33.34%) (Table 2) at 18 °C and highest mean RO (23.50) at 25 °C. Mean PPL, HR, and RO were lowest at 38 °C for Luzon, Visayas, and Mindanao (Additional file 1: Dataset S1).

**Table 2:** Descriptive statistics for PPL, HRs, and ROs of wet season-collected *Ae. aegypti* in the Philippine big islands by temperature

Big island	Temperature (°C)	PPL (%)		HRs (%)		ROs	
		Mean	SD	Mean	SD	Mean	SD
Luzon	18	15.73	1.43	39.29	6.20	24.67	2.42
	25	10.27	4.12	57.01	11.69	26.67	2.58
	38	7.33	2.85	2.50	3.39	1.17	1.33
Visayas	18	7.30	1.34	19.16	4.75	15.83	3.55
	25	6.41	0.49	27.44	5.22	20.17	0.98
	38	2.96	0.47	19.11	8.72	10.17	3.13
Mindanao	18	6.35	1.50	33.34	11.34	22.67	2.94
	25	6.79	1.48	30.31	10.71	23.50	3.94
	38	3.53	0.56	1.19	1.22	2.00	2.45
Philippines (overall mean)	18	9.79	24.89	30.59	62.76	21.06	26.33
	25	7.82	16.36	38.25	89.94	23.44	20.68
	38	4.61	13.96	7.68	53.70	4.58	25.84

### Development-related phenotypes of dry season-collected *Ae. aegypti*

Overall mean PPL and RO of dry season-collected *Ae. aegypti* were highest at 18 °C for both experimental (14.19% and 2.89, respectively) and control (15.82% and 3.33, respectively) photoperiods (Table 3). Overall mean HR was highest at 25 °C for both experimental and control (44.15% and 38.17%, respectively) photoperiods. Lowest mean PPL, HR, and RO were recorded at 38 °C for both control (7.57%, 2.12%, and 0.33, respectively) and experimental (8.86%, 2.70%, and 1.94, respectively) photoperiods. Within each big island, mean HRs of mosquitoes were highest at 25 °C and lowest at 38 °C for the experimental photoperiod (Table 3). Likewise, mean ROs were highest at both 18 and 38 °C for mosquitoes in the Visayas, at 18 °C for those in Mindanao, and at 25 °C for those in Luzon (Additional file 2: Dataset S2).

**Table 3:** Descriptive statistics for PPL, HRs, and ROs by photoperiod (control: 12L:12D; experimental: 13.5L:10.5D) and temperature of dry season *Ae. aegypti*

Big island	Photoperiod	Temperature (°C)	PPL (%)		HRs (%)		ROs	
			Mean	SD	Mean	SD	Mean	SD
Luzon	Control	18	16.72	12.50	34.40	22.65	3.17	1.51
		25	14.38	11.85	49.64	38.07	3.67	1.03
		38	5.85	12.65	4.17	20.41	0.17	0.82
	Experimental	18	14.29	13.57	37.50	43.25	3.50	1.10
		25	8.44	10.19	53.25	40.44	3.83	0.82
		38	10.61	3.83	3.08	8.74	1.00	2.53
Visayas	Control	18	16.35	11.81	31.01	62.80	3.33	1.63
		25	9.31	9.78	43.04	19.10	3.00	1.79
		38	8.61	7.49	2.19	8.52	0.83	2.34
	Experimental	18	14.26	16.94	38.04	62.80	2.67	3.50
		25	11.59	3.76	44.68	19.10	2.50	3.52
		38	7.49	5.54	2.94	8.52	2.67	3.27
Mindanao	Control	18	14.38	7.70	34.55	39.71	3.50	2.45
		25	11.89	11.47	21.82	33.78	2.67	1.03
		38	8.23	17.46	0.00	0.00	0.00	0.00
	Experimental	18	14.02	8.39	16.87	24.00	2.50	3.03
		25	10.83	8.54	34.52	44.48	2.00	3.10
		38	8.49	6.68	2.07	3.85	2.17	2.34
Philippines (average)	Control	18	15.82	5.22	33.32	15.95	3.33	0.91
		25	11.86	5.62	38.17	22.19	3.11	0.76
		38	7.57	6.32	2.12	6.17	0.33	0.77
	Experimental	18	14.19	6.31	30.80	23.93	2.89	1.37
		25	10.28	3.99	44.15	18.83	2.78	1.52
		38	8.86	2.90	2.70	3.50	1.94	1.47

### Full-factorial MANOVA results in both seasons of *Ae. aegypti*

The assumption of no multicollinearity among the dependent variables (PPL, HR, and RO) was satisfied in the given data set for MANOVA. The assumption of multicollinearity was not an issue among the independent variables of MANOVA because these categorical variables created independent groups upon which dependent variables were compared for significant differences. Results of the GLM for full-factorial MANOVA (Table 4) using Pillai’s trace, the most robust among SPSS tests (v. 21; IBM Corporation, NY, USA), showed no significant (*P* > 0.05) main effects of altitude on PPL, HR, or RO, but were significantly (*p* < 0.001) affected by the main effects of season, temperature, and big island. Moreover, significant interaction effects across all sites between (1) season and big island (*P* < 0.01), (2) season and temperature (*P* < 0.001), and (3) big island and temperature (*P* < 0.001), and among (1) season, big island, and temperature (*P* < 0.001), (2) big island, altitude, and temperature (*P* < 0.05), and (3) season, big island, altitude, and temperature (*P* < 0.05) were observed for all phenotypes. The mean temperatures were similar between the wet (min = 23.87 °C; max = 32.37 °C) and dry (min = 24.28 °C; max = 34.07 °C) seasons (Fig. 2) during the study period.

**Table 4:** MANOVA results using a generalized linear model for the main and interaction effects of big island, altitude, and temperature on PPL, HRs, and ROs between wet (2017) and dry (2018) season *Ae. aegypti* across all Philippine sites

Sources of variation	Pillai's trace	*F*	Hypothesis *df*	Error *df*	*P*-value
Main effects					
Season	0.959	971.809	3.000	124.0	** < 0.001**
Big island	0.380	9.781	6.000	250.0	** < 0.001**
Altitude	0.010	0.430	3.000	124.0	0.732
Temperature	0.993	41.103	6.000	250.0	** < 0.001**
Interaction effects					
Season × big island	0.179	4.086	6.000	250.0	**0.001**
Season × altitude	0.004	0.161	3.000	124.0	0.922
Season × temperature	0.888	33.276	6.000	250.0	** < 0.001**
Big island × altitude	0.042	0.885	6.000	250.0	0.506
Big island × temperature	0.685	9.314	12.000	378.0	** < 0.001**
Altitude × temperature	0.009	0.187	6.000	250.0	0.980
Season × big island × altitude	0.025	0.519	6.000	250.0	0.794
Season × big island × temperature	0.517	6.552	12.000	378.0	** < 0.001**
Season × altitude × temperature	0.044	0.928	6.000	250.0	0.475
Big island × altitude × temperature	0.194	2.180	12.000	378.0	**0.012**
Season × big island × altitude × temperature	0.161	1.783	12.000	378.0	**0.049**

Bold *P*-values mean significant at *P* = 0.05

### Overall full-factorial MANOVA results within season

#### Wet season-collected Ae. aegypti

GLM results of MANOVA within the wet season (Table 5) showed significant (*P* < 0.01) main effects of big island and temperature on PPL, HRs, and ROs of *Ae. aegypti*. The significant (*P* < 0.01) interaction effects were only observed between big island and temperature on all phenotypes.

**Table 5:** MANOVA results using a generalized linear model for the main and interaction effects of big island, altitude, and temperature on PPL, HRs, and ROs of wet season *Ae. aegypti* across all Philippine sites

Sources of variation	Pillai's trace	*F*	Hypothesis *df*	Error *df*	*P*-value
Main effects					
Big island	0.926	10.060	6.000	70.000	** < 0.001**
Altitude	0.005	0.061	3.000	34.000	0.980
Temperature	1.197	17.407	6.000	70.000	** < 0.001**
Interaction effects					
Big island × altitude	0.084	0.513	6.000	70.000	0.797
Big island × temperature	1.382	7.692	12.000	108.000	** < 0.001**
Altitude × temperature	0.225	1.479	6.000	70.000	0.198
Big island × altitude × temperature	0.397	1.371	12.000	108.000	0.191

Bold *P*-values mean significant at *P* = 0.05

#### Dry season-collected *Ae. aegypti*

Interestingly, the GLM of MANOVA results for dry season-collected *Ae. aegypti* (Table 6) were similar to those in the wet season. The main effects of big island (*P* < 0.05) and temperature (*P* < 0.01) on PPL, HRs, and ROs were significant. The significant (*P* < 0.01) interaction effects for dry season mosquitoes were detected only between temperature and photoperiod for both control and experimental photoperiods.

**Table 6:** MANOVA results using a generalized linear model for the main and interaction effects of big island, altitude and temperature on PPL, HRs, and ROs of dry season *Ae. aegypti* across all Philippine sites

Sources of variation	Pillai’s trace	*F*	Hypothesis *df*	Error *df*	*P*-value
Main effects					
Big island	0.226	3.019	6.000	142.000	**0.008**
Altitude	0.027	0.637	3.000	70.000	0.594
Temperature	0.940	20.984	6.000	142.000	** < 0.001**
Photoperiod	0.026	0.615	3.000	70.000	0.607
Interaction effects					
Big island × altitude	0.022	0.264	6.000	142.000	0.953
Big island × temperature	0.251	1.647	12.000	216.000	0.081
Big island × photoperiod	0.015	0.185	6.000	142.000	0.981
Altitude × temperature	0.043	0.519	6.000	142.000	0.793
Altitude × photoperiod	0.023	0.540	3.000	70.000	0.656
Temperature × photoperiod	0.260	3.529	6.000	142.000	**0.003**
Big island × altitude × temperature	0.078	0.483	12.000	216.000	0.923
Big island × altitude × photoperiod	0.111	1.387	6.000	142.000	0.224
Big island × temperature × photoperiod	0.256	1.679	12.000	216.000	0.073
Altitude × temperature × photoperiod	0.065	0.799	6.000	142.000	0.572
Big island × altitude × temperature × photoperiod	0.116	0.724	12.000	216.000	0.727

Bold *P*-values mean significant at *P* = 0.05

### Pharate larvae

#### Wet season

MANOVA results (Table 7) showed that the overall PPL of wet season *Ae. aegypti* across sites differed significantly (*P* < 0.01) by big island and temperature and not by altitude (*P* > 0.05). Results of Tukey’s post hoc test showed that the overall PPL across sites were significantly (*P* < 0.01) lowest at 38 °C and significantly highest at 18 °C (*P* < 0.05). A few surviving pharate larvae at 38 °C suggest their adaptive ability. Unhatched incompletely embryonated and severely desiccated eggs (Fig. 6c) were prevalent among those exposed at 38 °C.

**Table 7:** Results of MANOVA on the main and interaction effects of the Philippine big island, altitude, and temperature on PPL, HRs, and ROs of wet season *Ae. aegypti*

Source	*df*	PPL (%)		HRs (%)		ROs	
		*F*	*P*-value	*F*	*P*-value	*F*	*P*-value
Model	17	9.978	0.000	15.761	0.000	45.244	< 0.001
Intercept	1	708.559	0.000	604.826	0.000	2373.581	< 0.001
Big island	2	44.292	**0.000**	12.928	**0.000**	3.462	**0.042**
Altitude	1	0.050	0.825	0.161	0.690	0.150	0.701
Temperature	2	29.460	**0.000**	79.019	**0.000**	318.370	** < 0.001**
Big island × altitude	2	0.041	0.960	0.562	0.575	1.260	0.296
Big island × temperature	4	4.306	**0.006**	17.996	**0.000**	26.384	** < 0.001**
Altitude × temperature	2	0.600	0.554	1.567	0.223	0.407	0.669
Big island × altitude × temperature	4	0.891	0.479	1.911	0.130	4.118	**0.008**
Error	36						
Total	54						

Bold *P*-values mean significant at *P* = 0.05

#### Dry season

MANOVA results (Table 8) showed that only temperature had a significant (*P* = 0.00) main effect on PPL across all sites of dry season samples. Tukey’s post hoc analyses showed that PPL were significantly (*P* < 0.01) highest at 18 °C, and were not significantly different (*P* > 0.05) at 25 and 38 °C. Interaction effects between and among independent variables on PPL were not significant (*P* > 0.05).

**Table 8:** Results of MANOVA on the main and interaction effects of altitude, temperature and photoperiod on PPL, HRs, and ROs of dry season *Ae. aegypti* across the Philippine big islands

Source	*df*	PPL (%)		HR (%)		RO	
		*F*	*P*-value	*F*	*P*-value	*F*	*P*-value
Model	35	1.027	0.450	4.092	0.000	3.131	< 0.001
Big island	2	0.065	0.937	5.041	**0.009**	1.318	0.274
Altitude	1	0.113	0.737	1.302	0.258	1.119	0.294
Photoperiod	1	0.321	0.573	0.178	0.674	1.490	0.226
Temperature	2	12.314	**0.000**	53.678	**0.000**	30.801	** < 0.001**
Big island × altitude	2	0.170	0.844	0.475	0.624	0.086	0.918
Big island × photoperiod	2	0.065	0.937	0.145	0.865	0.139	0.870
Big island × temperature	4	0.111	0.978	1.255	0.296	3.245	**0.017**
Altitude × photoperiod	1	0.113	0.738	1.311	0.256	0.325	0.571
Altitude × temperature	2	0.369	0.693	0.947	0.393	0.881	0.419
Photoperiod × temperature	2	0.741	0.480	0.605	0.549	8.603	** < 0.001**
Big island × altitude × photoperiod	2	0.170	0.844	1.879	0.160	0.523	0.595
Big island × altitude × temperature	4	0.238	0.916	0.714	0.585	0.265	0.900
Big island × photoperiod × temperature	4	1.194	0.321	1.091	0.367	1.172	0.330
Altitude × photoperiod × temperature	2	0.280	0.757	0.300	0.742	1.040	0.359
Big island × altitude × photoperiod × temperature	4	0.221	0.926	0.508	0.730	0.285	0.887
Error	72						
Total	108						

Bold *P*-values mean significant at *P* = 0.05

### Hatch rates (HRs)

#### Wet season

Results of MANOVA (Table 7) revealed that the overall HRs of wet season *Ae. aegypti* F1 eggs across all sites differed significantly (*P* = 0.00) by main effects of both temperature and big island. The results of Tukey’s post hoc test showed that HRs were significantly (*P* < 0.001) lowest at 38 °C and significantly (*P* < 0.05) highest at 25 °C. There was also a significant (*P* < 0.001) interaction between big island and temperature that affected the HRs of F1 eggs across all sites. These findings constitute the first such report in the tropics, as most studies have been done in temperate and subtropical regions [35–38].

#### Dry season

Results of MANOVA (Table 8) showed that big island (*P* = 0.02) and temperature (*P* = 0.00) had significant main effects on HRs of dry season *Ae. aegypti* across all sites. The results of Tukey’s post hoc test showed that HRs were significantly (*P* < 0.05) highest at 25 °C, and were significantly (*P* < 0.001) lowest at 38 °C. No significant (*P* > 0.05) interaction effects were detected between and among big islands, altitude, temperature, and photoperiod on HRs of dry season-collected *Ae. aegypti*.

### Reproductive outputs (ROs)

#### Wet season

MANOVA results (Table 7) revealed that the overall ROs of wet season *Ae. aegypti* F1 egg cohorts across all sites differed significantly by main effects of big island (*P* < 0.05) and temperature (*P* < 0.01). The results of Tukey’s post hoc test showed that RO was significantly (*P* < 0.001) lowest at 38 °C and significantly (*P* < 0.05) highest at 25 °C. Significant interaction effects between big island and temperature (*P* < 0.01) and among big island, altitude, and temperature (*P* < 0.01) were observed on the ROs of wet season-collected *Ae. aegypti* egg cohorts across all sites.

#### Dry season

The results of MANOVA (Table 8) showed a significant main effect for temperature (*P* < 0.01) on ROs of dry season *Ae. aegypti* across the big islands. The results of Tukey’s post hoc test showed that ROs were significantly (*P* < 0.001) lowest at 38 °C, and did not significantly (*P* > 0.05) differ between 18 and 25 °C. Interaction effects between big island and temperature (*P* < 0.05) and between photoperiod and temperature (*P* < 0.01) significantly influenced the ROs of dry season *Ae. aegypti* across all big islands.

## Discussion

Results of full-factorial MANOVA in both seasons suggest that significant differences in *Ae. aegypti* phenotypes are not associated with season alone but rather their interaction with temperature and perhaps the associated factors of the Philippine big islands such as their ecology, available breeding sites, and day lengths due to latitudinal locations. The big islands represent different latitudes, with Mindanao being the closest to the equator (8°N), followed by Visayas (10°N), and then Luzon (14–16°N; Table 1). Latitudinal differences bring about variation in the amount of sunlight received at a location due to the angle of the sun’s rays [39]; thus, the photoperiod regulates the life history stages [40]. At 10° latitude, photoperiodism in insects has been observed [41]. Moreover, an increase in latitude results in a decrease in temperature [42], indicating that the sites farther from the equator with higher altitude have relatively lower temperature (Fig. 2) than those near the equator. Temperature greatly influences the mosquito population and disease transmission, as it directly affects the abundance of breeding sites, rate of mosquito development, reproduction, and survival [43, 44]. These interaction effects on *Ae. aegypti* suggest that their dynamics and apparent disease transmission require a careful evaluation of the existing vector and dengue control programs. Previously, Edillo et al. [45] reported a gradual increase in the minimum rate of dengue virus-infected *Ae. aegypti* in Cebu city, Philippines, from zero in wet months to 48.22 infected mosquitoes for every 1000 in mid-dry season (April), consistent with Angel and Joshi [46] and Thongrungkiat et al. [47]. Although control interventions were recommended in the non-outbreak dry season in order to suppress the re-emergence of dengue transmission in the next outbreak wet season, the common practice of the enhanced 4S strategy was conducted only during the epidemic wet season. The results of the current study suggest that the enhanced 4S strategy should be implemented year-round owing to the absence of seasonal differences in the PPL, HR, and RO of *Ae. aegypti* across the big islands as a consequence of global warming. Newly emerged pharate first-instar larvae of *Ae. aegypti* that had undergone prolonged quiescence were shown to be vulnerable to environmental stress [48]. Thus, targeting them year-round would greatly reduce the mosquito vector populations, with particular emphasis on Visayas at 10°N latitude [41] and Mindanao at 8°N latitude.

Climate change-induced increased temperature and rainfall variability are likely to have the greatest impacts on human health in the Philippines [49, 50]. This scenario might be exacerbated in rural highlands that do not have direct water pipelines, forcing households to store water in plastic drums for domestic use. This lack of a proper water service system facilitates year-round breeding of mosquitoes in their expanded distribution to rural highlands, as also confirmed in our separate study on the population genetics of Philippine *Ae. aegypti* based on gene flow, posing a challenge for vector control programs.

Pharate larvae of *Ae. aegypti* were highest at 18 °C in both seasons in the current study, which is consistent with the results reported by Farnesi et al. [31]. Moreover, Clements [34], Diniz et al. [14], and Vinogradova [13] noted that as pharate larvae of *Ae. aegypti* are subjected to unfavorable environmental conditions (i.e., temperature change), temporary developmental arrest and impeded larval hatching follow, which is consistent with the current results. Diapausing pharate larvae of *Ae. albopictus* may be more metabolically active at 21 °C than at lower temperatures and spend considerable time at higher temperatures during fall before the onset of winter [11]. The current results corroborate those of related studies [51–53] showing that desiccation resistance of these eggs is an important ecological trait that might be associated with their adaptation to drought tolerance, invasion success towards higher altitudes, and competitive outcomes. Moreover, quiescence depends on differences in eggshell composition and structural and physiological changes, which result in reduced metabolism among pharate larvae contained within the egg chorion [14]. The eggs, not the embryos, are able to resist desiccation, because quiescence can only be initiated after completed embryogenesis, hence the term “egg resistance to desiccation.” The chitin synthase gene promotes chitin synthesis, secreted into the egg’s extracellular space, which leads to the formation of the serosal cuticle for egg desiccation resistance [14, 54]. The high amount of fatty acyl-CoA elongase in mature oocytes of insects produces hydrocarbons to regulate water loss; its abundance varies in temperate *Ae. albopictus* exposed to long and short days but is maintained at a relatively constant amount in tropical populations [55]. Poelchau et al. [11] found that the gene expression of diapausing *Ae. albopictus* eggs converged over time towards quiescence. Metabolic differences, particularly in lipid storage, serve as the primary distinguishing factor between gene expression of early diapause and quiescence. Exogenous control of quiescence denotes its non-reliance on rapid gene activation and macromolecule synthesis or degradation [14]. Quiescence begins when the embryo (pharate larva) receives an external unfavorable stimulus (i.e., rapid change in humidity and/or temperature), whereas gravid females initiate the expression of certain genes to be transferred to the offspring, and the embryo enters diapause. Egg quiescence is temporary and reversible, and has been shown in other mosquito species such as *Ae. flavopictus*, *Ae. galloisi*, *Ae. riversi*, *Anopheles aquasalis*, *An. gambiae*, and *Culex quinquefasciatus* [14].

Overall, the mean HRs of *Ae. aegypti* were highest at 25 °C and lowest at 38 °C in both the wet season (2017–2018) and dry season (2018), and were significantly influenced by temperature and big island with associated day lengths due to latitudinal differences (Table 1). During the wet season, mean HRs were highest at 25 °C for mosquitoes in Luzon and Visayas and at 18 °C for those in Mindanao. During the dry season, mean HRs were highest at 25 °C for all mosquitoes in all big islands. *Aedes aegypti* eggs from highlands that were exposed at 38 °C and hatched into larvae imply their apparent gradual adaptation as they expand their distribution to higher altitude, i.e., up to 1489 m ASL in BG sub-sites. The range of temperature tolerance for *Ae. aegypti* is between 10 °C [32, 56–58] and 32 °C [58], but up to 35 °C for short periods of flight [57] and maximum tolerance for embryonic development [31]. Egg viability between 16 and 31 °C was above 80%, comparable to *Ae. aegypti* presence in the tropics and subtropics [31]. Likewise, Dhimal et al. [10] found *Ae. aegypti* and *Ae. albopictus* up to 1350 m ASL in Katmandu Valley, Nepal, and a few between 1750 m and 2100 m ASL in the mountains. Moreover, results were consistent with the findings of De Majo et al. [38], who reported that the proportion of hatched eggs of *Ae. aegypti* in Argentina was positively associated with immersion and pre-immersion temperature and photoperiod. Lacour et al. [22] exposed both tropical and temperate *Ae. albopictus* to short-day and long-day photoperiods, and found that only the eggs of temperate strains maternally reared under short days entered diapause, with an HR of 0.1–13.6%.

RO results indicate the plasticity of *Ae. aegypti* as they adapt to their local weather conditions across the Philippine big islands during the hotter, dry and longer day lengths in summer [59, 60]. During the dry season with experimental (longer) photoperiod (Table 3), ROs of mosquitoes in Visayas (10° N latitude) ranged from 2.5 to 2.67, and those in Mindanao (8°N latitude) from 2 to 2.5, in all three temperatures, but for those in Luzon, mean RO (3.83) was highest at 25 °C and lowest at 38 °C (1.0). RO results in Visayas and Mindanao were consistent with the findings of Denlinger [41], who reported that several insect species show photoperiodism because seasonal changes in day length are greater within 10°N of the equator. Moreover, a larger diurnal temperature range in the highlands during the dry season might also have contributed to the reduction in RO, consistent with results reported by Carrington et al. [60]. Overall, mean ROs across the big islands were lowest at 38 °C in both seasons, consistent with findings of Bar-Zeev [61]. Hence, temperature can dictate optimal conditions for arbovirus emergence and spread, and is considered a strong driver of transmission of vector-borne diseases.

In summary, we hypothesized that temperature, season, photoperiod as influenced by latitudinal differences, and altitude might play a role in the development-related phenotypes of *Ae. aegypti* F1 eggs. Three prevailing results for *Ae. aegypti* in both wet (2017–2018) and dry (2018) seasons across all sites were observed: (1) significant main effects of temperature, season, and big island on all phenotypes (PPL, HRs, and ROs), and (2) significant interaction effects on all the phenotypes of *Ae. aegypti* between the following: (i) season and big island, (ii) season and temperature, (iii) season, big island, and temperature, (iv) big island, altitude, and temperature, and (v) season, big island, altitude, and temperature. (3) Hence, season alone did not influence the phenotypic differences in *Ae. aegypti* because the average temperatures were similar between the two seasons of the study period (Fig. 2). Temperature not only affects quiescence directly by triggering it as an acyclic environmental change, but it also indirectly affects the initiation of dormancy by inhibiting complete embryogenesis [31]. The larval development time is significantly prolonged and lipid reserves are decreased, incurring fitness costs for larval viability and compromising adult mosquitoes’ reproductive performance [13, 34]. Quiescence directly affects the survival of mosquito populations under adverse environmental conditions [13, 62]. The environmental context of climate change and global warming is likely to contribute to the spread of *Ae. aegypti* and its pathogens in new areas [63].

Gould and Higgs [64] reported that climate is a major factor in arboviral evolution and transmission efficiency from arthropod vectors to vertebrate hosts such as humans. Viruses vary seasonally. In the case of dengue, virus isolation and detection in *Ae. aegypti* is higher during the dry season than in the wet season [45]. Transmission of dengue among humans is higher during the wet season, most especially during the early months of the wet season. Latitude and altitude associated with highlands and lowlands may influence the effects of critical photoperiod (CPP) among *Ae. aegypti* populations. This current study provides the link to related studies of *Ae. sierrensis* and *Ae. triseriatus* that show these combined effects [65, 66]. Denlinger and Armbruster [12] suggested that the rapid evolution of the CPP in *Ae. albopictus* across the spatial climatic gradient of eastern North America may imply that mosquitoes quickly adjust their phenology (i.e., the cyclic and seasonal natural phenomena in relation to climate) to expand their geographic range and make the most of extended seasons associated with global warming.

## Conclusions

In conclusion, development-related phenotypes (PPL, HRs, and ROs) of *Ae. aegypti* across Philippine study sites were significantly affected by temperature, season, and their latitudinal locations (i.e., big islands from 8°N to 16°N) in wet and dry seasons of 2017–2018. Season alone did not influence the phenotypes between seasons, but rather its interaction effects with big island and/or temperature, and/or altitude. Pharate larval formation of *Ae. aegypti* via egg quiescence in cold and hot conditions and their resistance to desiccation are important phenotypic adaptations. The capacity of mosquito eggs to survive for long periods has implications for the control of mosquitos and their potential to transmit human diseases. Thus, we recommend that a dengue prevention and control program in the Philippines, particularly the enhanced 4S strategy, be implemented year-round rather than during the dengue epidemic wet season, with a particular focus in the Visayas and Mindanao. Reduction of breeding sites, covering of water storage containers, and hygiene and sanitation around households should be constant components of a community-based, integrated approach, combined with educational programs to increase knowledge and understanding of best practice [67]. Also, we recommend the installation of pipelines for the water supply system for householders in rural highlands to decrease potential breeding sites. Thus, the findings of this study can guide dengue and vector control programs to better respond to the effects of global warming, not just in the Philippines, but in the tropics in general.

## Supplementary Information

**Additional file 1: Dataset S1.** Development-related phenotypes (PPL, HR and RO) of *Aedes aegypti* for wet season (2017–2018).

**Additional file 2: Dataset S2.** Development-related phenotypes (PPL, HR and RO) of *Aedes aegypti* for dry season (2018).

## Data Availability

The meteorological data that support the findings of this study are available from the Philippine Atmospheric, Geophysical and Astronomical Services Administration (PAGASA) and were requested through the online portal of the Philippine Freedom of Information (foi.gov.ph) but restrictions apply to the availability of these data, which were used under specific terms and conditions for the current study, and so are not publicly available. Data are, however, available from the authors upon reasonable request and with permission of PAGASA. Datasets for development-related phenotypes (percent pharate larvae, hatching rate and reproductive output) of *Ae. aegypti* for wet (2017–2018) and dry (2018) seasons analyzed in this study are included in this published article and its supplementary information files.

## Abbreviations

PPL: Percent pharate larvae
HR: Hatching rate
RO: Reproductive output
RH: Relative humidity
NaOCl: Sodium hypochlorite
EDTA: Ethylenediaminetetraacetic acid
DW: Distilled water
CRD: Completely randomized design
GLM: Generalized linear model
MANOVA: Multivariate analyses of variance
DOH: Department of Health
PAGASA: Philippine Atmospheric, Geophysical and Astronomical Services Administration
CPP: Critical photoperiod
